# A hierarchical risk assessment framework for head-to-head comparison of statin safety profiles in Chinese patients

**DOI:** 10.1038/s41598-026-47279-y

**Published:** 2026-04-09

**Authors:** Leo Tsui, Dan Wang, Chuyun Fan, Chengling Shi

**Affiliations:** 1 College of Bee Science and Biomedicine, Fujian Agriculture and Forestry University, Fuzhou, 350002 China; 2 School of Pharmacy, Shanghai University of Medicine and Health Sciences, Shanghai, 200237 China; 3 Department of Pharmacy, PLA Navy Characteristic Medical Center, Shanghai, 200052 China

**Keywords:** Hierarchical risk assessment framework, Myopathy, Hepatotoxicity, Statins, Drug safety, Diseases, Drug discovery, Medical research, Risk factors

## Abstract

Ethnic variations in lifestyle, diet, and pharmacogenetics significantly influence statin tolerance. Extending our previous work that documented overall ADR incidence, this study introduces a Hierarchical Risk Assessment Framework (HRAF) to systematically compare statin safety profiles through multi-layered statistical analysis and network visualization. We conducted a post-hoc pooled analysis of Chinese clinical trials (1992–2023) involving seven statins. Our triple-analytical HRAF comprised: (1) global 2 × 7 contingency analysis using Chi-squared tests; (2) individual statin risk profiling with Bonferroni-corrected pairwise comparisons against aggregate controls; and (3) comprehensive pairwise comparisons with Tukey adjustment and network visualization. Muscle-related (189 study units; *n* = 31,763) and liver-related (188 study units; *n* = 31,281) adverse drug reactions were analyzed. Applying the HRAF consistently revealed distinct statin-specific safety hierarchies. Global tests confirmed significant class heterogeneity (both *p* < 0.001). Individual profiling identified atorvastatin with elevated myopathy risk (1.78%; Bonferroni *p* < 0.001) and pitavastatin with pronounced hepatotoxicity (5.36%; Bonferroni *p* < 0.001) alongside significant myopathy risk (2.53%; Bonferroni *p* = 0.002). Pairwise comparisons established detailed risk gradients: atorvastatin showed higher myopathy risk versus five statins (all Tukey *p* < 0.001 except pitavastatin), while pitavastatin demonstrated superior hepatotoxicity risk versus all comparators (all Tukey *p* < 0.001) and significant myopathy risk versus multiple statins. Network analysis visually confirmed these high-risk statins as central nodes with multiple significant connections for both ADR types. Using this proposed HRAF provides robust, quantitative safety data for statins in Chinese patients, supporting risk-stratified prescribing that pitavastatin warrants heightened caution regarding liver function, while atorvastatin should be used judiciously in myopathy-susceptible patients. Rosuvastatin and simvastatin emerge as lower-risk alternatives for both outcomes.

**Supplementary Information:**

The online version contains supplementary material available at 10.1038/s41598-026-47279-y.

## Introduction

Statins, while cornerstone therapies for cardiovascular prevention, are hampered by a class-wide risk of myopathy and hepatotoxicity, leading to significant non-adherence and treatment discontinuation^1,2^. The Chinese population, characterized by dietary patterns consistent with traditional Asian diets and distinct genetic variations in lipid metabolism compared to Western Caucasian populations, provides a unique framework for investigating the safety profiles of statins^3^. Due to these unique characteristics of the Chinese population, the maximum approved doses for some statins in Asian nations, such as Japan and China, are set at lower levels than those in other countries^4,5^. The HPS2-THRIVE study indicates a notable rise in elevated alanine aminotransferase (ALT) levels among Chinese participants, emphasizing the necessity of liver function monitoring^6^. Additionally, muscle symptoms, a major cause of statin therapy discontinuation, are prevalent in the Chinese population^2^. Given that liver enzyme elevations are dose-dependent and influenced by genetic and ethnic factors, vigilant monitoring of both liver- and muscle-related ADRs is crucial for safe and effective treatment in the Chinese population.

While our prior systematic review documented the incidence of muscle and liver ADRs in Chinese trials from 1992 to 2023^7^, it could not provide the head-to-head comparative analysis needed by clinicians and policymakers to navigate statin-specific risks^8,9^. Therefore, to bridge this decisional gap, we performed a post-hoc analysis of Chinese clinical trials to conduct a head-to-head comparison of seven statins. Utilizing a Hierarchical Risk Assessment Framework (HRAF)—encompassing global association tests, individual risk profiling, and comprehensive pairwise comparisons—this study aims to definitively rank statin drugs by their myopathic and hepatotoxic risks, delivering the actionable insights required to personalize statin therapy for Chinese patients.

## Methods

### Data sources

**Fig. 1:**
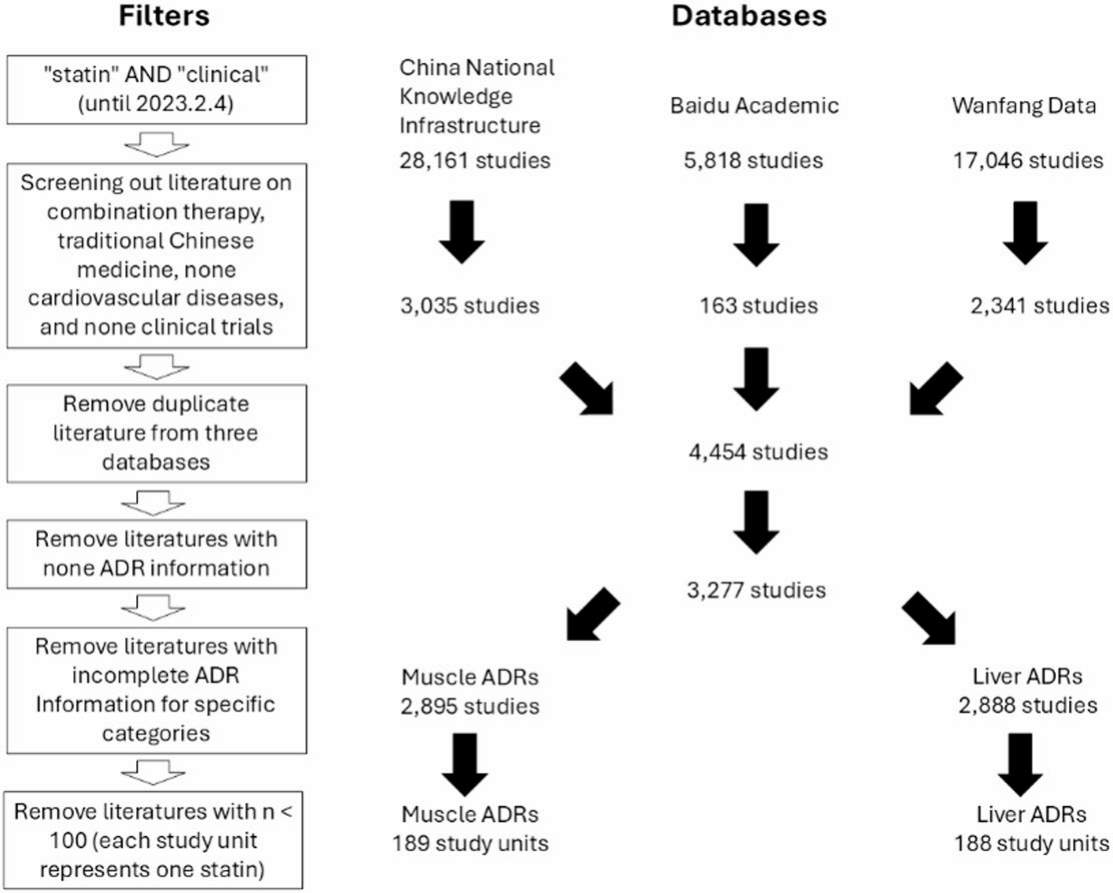
Conducting an adverse drug reactions (ADRs) database based on clinical trials with statin therapy from Chinese journals.

The ADR data were sourced from a systematic literature search conducted in our previous study^7^. We searched three Chinese databases, including China National Knowledge Infrastructure (CNKI), Wanfang Data Knowledge Service Platform, and Baidu Scholar, from 1992 to February 4, 2023. In brief, Wanfang used the keyword search criteria “Abstract: ‘Statin’ AND ‘Clinical’”; CNKI Data employed “Abstract: (‘Statin’ AND ‘Clinical’) AND Title or Keyword: (‘Statin’ AND ‘Clinical’)”; while Baidu Scholar directly searched for “’Statin’ AND ‘Clinical’”. The search yielded a total of 51,025 articles from 1992 to 2023, with 28,161 articles from CNKI, 17,046 from Wanfang, and 5,818 from Baidu Scholar (Fig. 1).

### Inclusion and exclusion criteria

This study analyzed statin-related ADRs in Chinese adults with cardiovascular diseases (CVDs) using data from clinical trials that reported original statin interventions with detailed outcomes. To enable a direct head-to-head comparison of statin safety, we exclusively included trials where patients received statin monotherapy. For studies involving combination therapies, only data unequivocally attributable to statins were extracted. Studies were excluded if ADRs could not be clearly linked to statins, if they lacked complete ADR or patient data, or if the full text was unavailable.

### Study selection

We screened 51,025 articles using a four-phase process to ensure objectivity and accuracy (Fig. 1). Each article was reviewed by two independent reviewers and verified by a third inspector. First, irrelevant studies (e.g., non-CVD, combination therapies, traditional Chinese medicine, or non-clinical trials) were excluded, yielding 5,539 clinical trials from CNKI, Wanfang, and Baidu Scholar. After deduplication, 4,454 unique studies remained. Full-text screening removed articles lacking ADR data, reducing the pool to 3,277. Finally, after excluding studies with incomplete or unclear muscle/liver ADR data, 2,895 muscle-related and 2,888 liver-related studies were included in the analysis.

### Data extraction

The ADRs were defined as harmful responses occurring at normal statin doses during disease prevention, diagnosis, or treatment, unrelated to therapeutic intent. Adverse events or cardiovascular events lacking a clear causal link to statin use were excluded from the analysis. For liver and muscle ADR assessments, we extracted three key data columns: “Total ADR cases,” “Liver/muscle ADR cases,” and “Total cases.” Since most clinical trials did not report ADR severity, this factor was omitted from the analysis.

### Threshold setting and trial selection

Detecting rare ADRs in small-sample trials is limited by low statistical power. Surprisingly, our analysis of large-scale trials failed to identify rare muscle- or liver-related ADRs previously reported in smaller studies, suggesting potential publication bias^10^. To improve detection and reduce bias, we focused on trials with ≥ 100 participants and applied a 1.0% threshold for both muscle and liver ADRs, based on prior data (muscle ADR incidence: 0.951%)^7^. For head-to-head comparisons, each eligible statin arm was treated as an independent study unit, with multi-arm trials split accordingly. This yielded 189 muscle-related and 188 liver-related study units for analysis, enhancing robustness and reliability of statin safety evaluations (Fig. 1).

### The hierarchical risk assessment framework

To ensure rigorous and progressively validated findings in head-to-head drug safety comparisons, we designed a three-tiered HRAF. This framework comprises: (1) global association testing to detect overall differences among statins; (2) individual risk profiling to identify statins with aberrant safety profiles; and (3) pairwise comparisons to validate and establish detailed risk hierarchies. This three-tiered design ensures progressively validated findings—with each layer building upon and corroborating the previous one. Global detection identifies whether any differences exist, individual profiling pinpoints which statins drive these differences, and pairwise confirmation quantifies the precise risk relationships between all statins. This interdependent, multi-layered approach substantially strengthens the robustness and reliability of the final risk hierarchies for clinical decision-making. In this study, we performed a post-hoc analysis of ADR data for seven statins using this framework specifically designed for head-to-head statin safety comparisons in Chinese patients.

Tier 1: Global Association Testing. A global 2 × 7 contingency table analysis using Pearson’s Chi-squared test was conducted to assess the overall association between statin treatment and ADR incidence, revealing highly significant associations for both muscle and liver ADRs.

Tier 2: Individual Risk Profiling. To identify statins with aberrant risk profiles, we performed comparisons using 2 × 2 contingency tables wherein each statin’s ADR incidence was tested against the aggregated incidence of the other six statins combined using Chi-squared tests. These 2 × 2 tests were conducted without Yates’ continuity correction to maintain statistical power, and a Bonferroni correction was applied to this family of 7 tests per ADR type, setting the significance threshold at p-values < 0.007 (0.05/7 comparisons). The Bonferroni method was selected for this focused set of comparisons due to its conservative nature and straightforward interpretation when testing individual statins against the aggregate reference. Additionally, standardized residuals were examined from the overall tests.

Tier 3: Pairwise Comparison and Risk Hierarchy Establishment. To delineate the specific risk hierarchy between all individual statins, we conducted post-hoc pairwise comparisons with p-values adjusted using the Tukey method. Tukey’s Honest Significant Difference (HSD) was chosen for the comprehensive pairwise comparisons as it is specifically designed for all-pairwise testing in Analysis of Variance (ANOVA) and Generalized Linear Model (GLM) frameworks, providing better statistical power while maintaining strong family-wise error rate control.

### Statistics

All analyses were conducted separately for muscle and liver ADRs using R software, using the emmeans package for pairwise comparisons. Heatmap visualizations of all pairwise comparisons were generated using the ggplot2 package, displaying Tukey-adjusted p-values with color gradients. In each heatmap cell, red indicates that the column statin has a significantly higher ADR incidence than the row statin, and blue indicates the opposite. Asterisks denote statistical significance (* *p* < 0.05, ** *p* < 0.01, *** *p* < 0.001). Network plots based on statistically significant pairwise comparisons (Tukey-adjusted *p* < 0.05) from Tables 2 and 3 were generated using the igraph package to provide intuitive representations of the risk hierarchies. In these directed networks, arrows point from the higher-risk statin to the lower-risk statin, providing unambiguous direction of risk differences. Node sizes are proportional to ADR incidence rates, and network centrality metrics (degree and betweenness) were calculated to identify key statins. Edge thickness reflects the magnitude of the estimated difference. Network plots were created using the Fruchterman-Reingold layout algorithm with 10,000 iterations for optimal node placement, with separate legends for clarity.

## Results

To systematically compare statin safety profiles in Chinese patients, we applied the HRAF to muscle and liver ADR data from large-scale clinical trials (*n* ≥ 100). Table 1 summarizes the post-hoc analysis results. For muscle ADRs, the total number of ADR-positive cases was 355 out of 31,763 from 189 study units (Table 1; Fig. 1). In terms of liver ADRs, the overall number of ADR-positive cases was 382 out of 31,281 from 188 study units (Table 1; Fig. 1).

### Global association testing

To investigate whether there are differences in the positive rates of muscle and liver ADRs across various statin drugs, chi-squared tests were conducted comparing muscle- and liver-related ADRs. The analysis used the same contingency table structure, with degrees of freedom remaining consistent at 6 for both tests, suggesting similar categorical breakdowns (Table 1). The muscle ADRs analysis yielded a chi-squared statistic of 102.98 (df = 6, p-value < 2.2e^− 16^), while the liver ADRs analysis showed a chi-squared statistic of 180.51 (df = 6, p-value < 2.2e^− 16^). Both results were highly statistically significant, indicating strong evidence against the null hypothesis of no association between statin drug type and the respective ADRs.

### Individual risk profiling

Following a significant overall test, we performed seven Chi-squared tests (one per statin; Table 1), comparing the incidence in each statin against the aggregated incidence of all other six statins, for each ADR type. A Bonferroni correction was applied to each family of 7 tests, setting the significance threshold at a p-value < 0.007. For muscle-related ADRs, the incidence in patients receiving atorvastatin (1.78%) was significantly higher than the aggregate of other statins (*p* < 0.001). Conversely, the incidence was significantly lower for both rosuvastatin (0.71%, *p* < 0.001) and simvastatin (0.48%, *p* < 0.001). The incidence for pitavastatin (2.53%) was also elevated, reaching statistical significance after Bonferroni correction (*p* = 0.002). A more pronounced risk profile was observed for liver-related ADRs. Pitavastatin demonstrated a markedly high incidence of hepatotoxicity at 5.36%, which was significantly greater than the comparison group (*p* < 0.001). Atorvastatin also showed a significantly higher incidence (1.82%, *p* < 0.001). Mirroring the findings for myopathy, both rosuvastatin (0.54%, *p* < 0.001) and simvastatin (0.62%, *p* < 0.001) were associated with a significantly lower incidence of liver ADRs. The ADR rates for fluvastatin, pravastatin, and lovastatin did not show statistically significant differences from the aggregate incidence of the other statins for either muscle or liver outcomes after correction for multiple comparisons (all *p* > 0.007).

### Examination of residuals

To further validate the individual risk profiling results and identify which specific statins drove the overall associations, we examined standardized residuals. For muscle ADRs, atorvastatin demonstrated a strong positive association (residual = 9.05), while rosuvastatin and simvastatin showed strong negative associations. Pitavastatin was also associated with a higher frequency of muscle ADRs, and pravastatin approached the significance threshold (absolute residual > 2; Supplemental Table 1). The pattern was more pronounced for liver ADRs, where pitavastatin exhibited an exceptionally large positive residual (9.73), indicating a strong association with hepatotoxicity. Atorvastatin also showed a significant positive residual (7.45), whereas rosuvastatin and simvastatin were negatively associated (Supplemental Table 2). This residual analysis confirms that the significant overall associations are primarily driven by a higher risk profile for atorvastatin (muscle and liver) and pitavastatin (notably liver), alongside a lower risk profile for rosuvastatin and simvastatin.

### Pairwise comparisons

Having identified high-risk and low-risk statins through individual profiling, we proceeded to the third tier of the HRAF, pairwise comparisons, to establish the complete risk hierarchy between all statins. For muscle-related ADRs, two statins demonstrated a consistently higher risk profile. Atorvastatin showed significantly higher incidence rates compared to five of the six other statins (all *p* < 0.001). Pitavastatin, despite a smaller sample size, also exhibited significantly higher rates than most comparators, with all pairwise *p* < 0.0001 except when compared to atorvastatin (*p* = 0.0120). Conversely, rosuvastatin and simvastatin formed a lower-risk group, with simvastatin in particular showing a significantly lower incidence than multiple other statins. The remaining statins (fluvastatin, lovastatin, pravastatin) occupied an intermediate risk position, with few significant differences among themselves (Table 2).

For liver-related ADRs, the risk hierarchy was even more pronounced. Pitavastatin demonstrated a uniquely elevated risk, with its incidence being significantly higher than every other statin (all *p* < 0.0001). Atorvastatin again showed a higher-risk profile, with a significantly greater incidence than four other statins. The lower-risk group comprised rosuvastatin and simvastatin, which did not differ significantly from each other but consistently showed lower rates than the higher-risk statins. The profile of fluvastatin, lovastatin, and pravastatin was more variable for liver ADRs, though pravastatin generally trended towards the lower end of the spectrum (Table 3).

**Table 1:** Pooled incidence of statin-associated adverse drug reactions (ADRs) from Chinese clinical trials (sample size ≥ 100).

Statin	Muscle ADRs			Liver ADRs		
	Total patients	*n* (%)	*p*-value vs. total*	Total patients	*n* (%)	*p*-value vs. total*
Pitavastatin	553	14 (2.53)	**0.001**	653	35 (5.36)	**< 0.001**
Atorvastatin	12,456	222 (1.78)	**< 0.001**	11,707	213 (1.82)	**< 0.001**
Fluvastatin	1,444	14 (0.97)	0.584	1,444	22 (1.52)	0.284
Pravastatin	1,690	9 (0.53)	0.019	1,690	16 (0.95)	0.291
Lovastatin	1,350	10 (0.74)	0.178	1,350	13 (0.96)	0.377
Rosuvastatin	7,611	54 (0.71)	**< 0.001**	7,778	42 (0.54)	**< 0.001**
Simvastatin	6,659	32 (0.48)	**< 0.001**	6,659	41 (0.62)	**< 0.001**
Overall	31,763	355 (1.12)	-	31,281	382 (1.22)	-

* P-values were derived from Chi-squared tests, comparing each statin’s ADR rate against the pooled incidence of all other statins combined. Significance was evaluated against a Bonferroni-corrected threshold of a p-value < 0.007. Bolded p-values indicate statistical significance after correction.

**Table 2:** Pairwise comparisons of muscle-related adverse drug reactions (ADRs) between statin drugs. The estimated difference in effect between the two drugs, and positive values indicate higher muscle ADRs for the first drug listed. *, p-value < 0.05; **, p-value < 0.01; ***, p-value < 0.001.

Pair-wised statin ADRs	Estimated differences	*p*-value
Atorvastatin - Fluvastatin	0.6171	***<0.0001
Atorvastatin - Lovastatin	0.8885	***<0.0001
Atorvastatin - Pitavastatin	-0.3586	*0.0120
Atorvastatin - Pravastatin	1.2206	***<0.0001
Atorvastatin - Rosuvastatin	0.9319	***<0.0001
Atorvastatin - Simvastatin	1.3239	***<0.0001
Fluvastatin - Lovastatin	0.2715	0.5975
Fluvastatin - Pitavastatin	-0.9757	***<0.0001
Fluvastatin - Pravastatin	0.6035	** 0.0037
Fluvastatin - Rosuvastatin	0.3149	0.0829
Fluvastatin - Simvastatin	0.7068	***<0.0001
Lovastatin - Pitavastatin	-1.2472	***<0.0001
Lovastatin - Pravastatin	0.3321	0.4761
Lovastatin - Rosuvastatin	0.0434	0.9999
Lovastatin - Simvastatin	0.4353	* 0.0257
Pitavastatin - Pravastatin	1.5793	***<0.0001
Pitavastatin - Rosuvastatin	1.2906	***<0.0001
Pitavastatin - Simvastatin	1.6825	***<0.0001
Pravastatin - Rosuvastatin	-0.2887	0.3434
Pravastatin - Simvastatin	0.1033	0.9913
Rosuvastatin - Simvastatin	0.3919	***<0.0001

**Table 3:** Pairwise comparisons of liver-related adverse drug reactions (ADRs) between statin drugs. The estimated difference in effect between the two drugs, and positive values indicate higher liver ADRs for the first drug listed. *, p-value < 0.05; **, p-value < 0.01; ***, p-value < 0.001.

Pair-wised statin ADRs	Estimated differences	*p*-value
Atorvastatin - Fluvastatin	0.1805	0.3431
Atorvastatin - Lovastatin	0.6449	***<0.0001
Atorvastatin - Pitavastatin	-1.1171	***<0.0001
Atorvastatin - Pravastatin	0.6621	***<0.0001
Atorvastatin - Rosuvastatin	1.2277	***<0.0001
Atorvastatin - Simvastatin	1.0957	***<0.0001
Fluvastatin - Lovastatin	0.4645	**0.0087
Fluvastatin - Pitavastatin	-1.2976	***<0.0001
Fluvastatin - Pravastatin	0.4816	**0.0022
Fluvastatin - Rosuvastatin	1.0472	***<0.0001
Fluvastatin - Simvastatin	0.9152	***<0.0001
Lovastatin - Pitavastatin	-1.7621	***<0.0001
Lovastatin - Pravastatin	0.0171	1.0000
Lovastatin - Rosuvastatin	0.5827	***<0.0001
Lovastatin - Simvastatin	0.4507	**0.0036
Pitavastatin - Pravastatin	1.7792	***<0.0001
Pitavastatin - Rosuvastatin	2.3448	***<0.0001
Pitavastatin - Simvastatin	2.2128	***<0.0001
Pravastatin - Rosuvastatin	0.5656	***<0.0001
Pravastatin - Simvastatin	0.4336	**0.0021
Rosuvastatin - Simvastatin	-0.1320	0. 6914

**Fig. 2:**
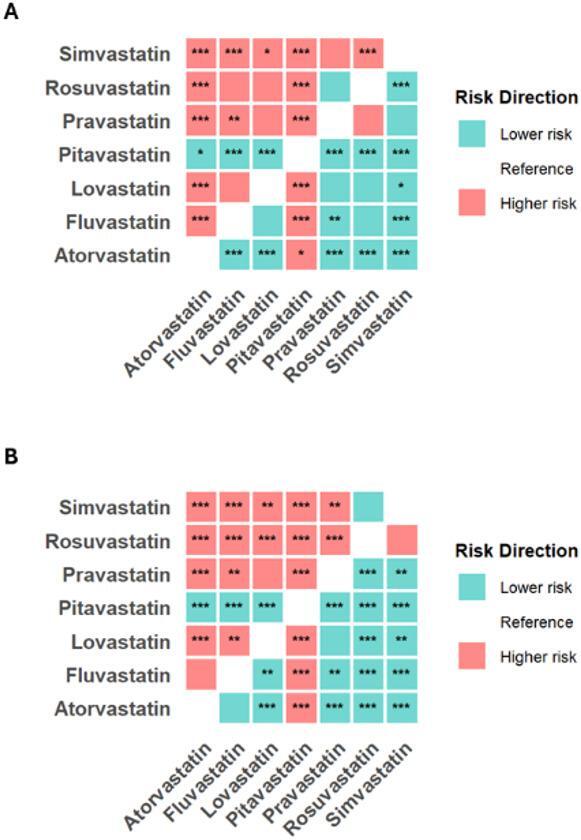
Heatmap visualization of pairwise comparisons for (**A**) muscle and (**B**) liver adverse drug reactions (ADRs) among seven statins. Each cell represents the Tukey-adjusted p-value for the comparison between the column statin and the row statin. The color scale indicates risk direction: red signifies that the column statin has a significantly higher ADR incidence than the row statin; blue signifies that the column statin has a significantly lower incidence than the row statin. Asterisks denote statistical significance after Tukey adjustment (* *p* < 0.05, ** *p* < 0.01, *** *p* < 0.001).

To sum up, heatmap visualizations complemented the tabular results by providing comprehensive overviews of all pairwise comparisons, with color gradients representing effect sizes and stars denoting statistical significance after Tukey adjustment (Fig. 2A, B).

### Network analysis of statin-specific ADR profiles

**Fig. 3:**
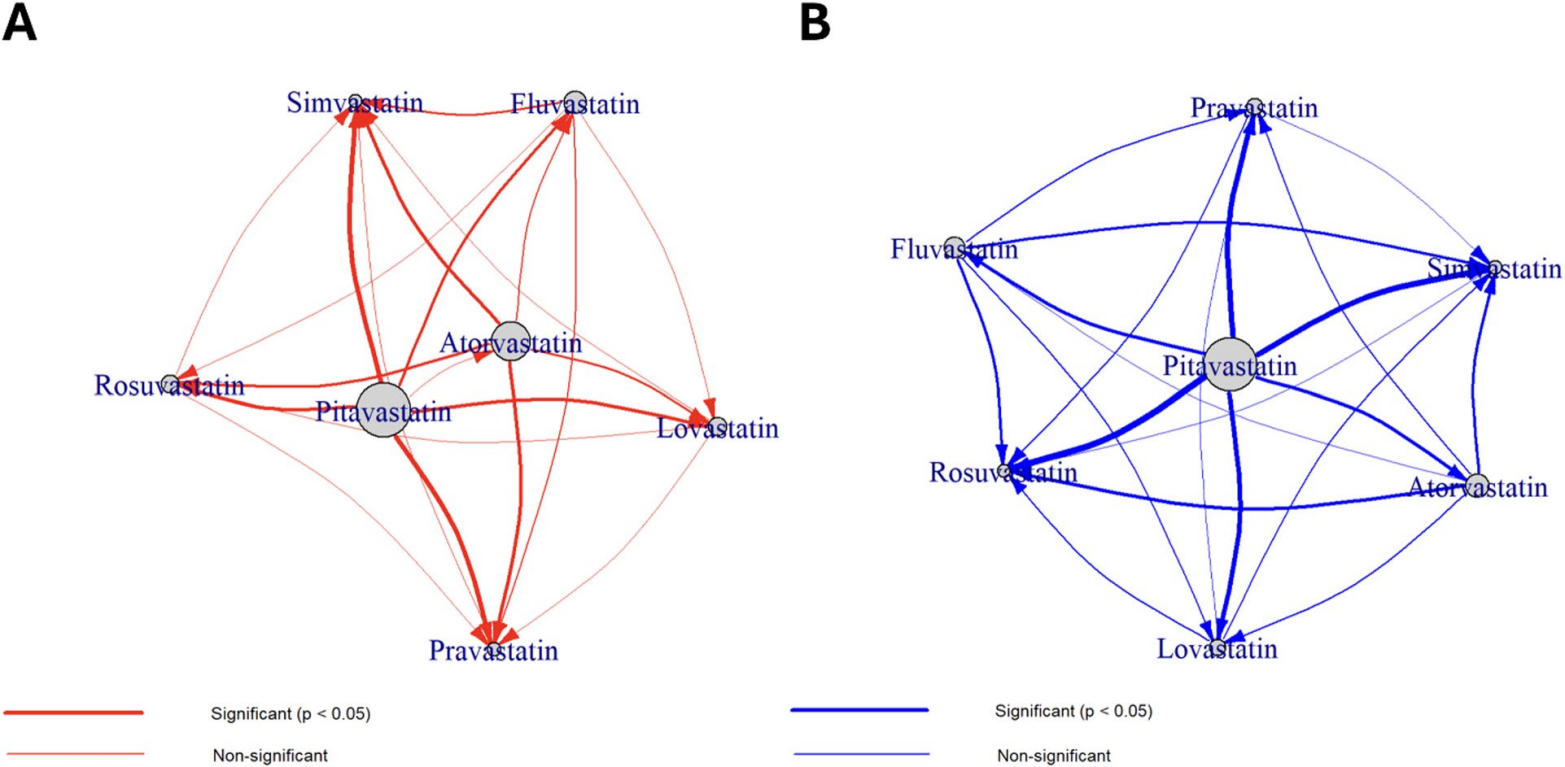
Network analysis of significant pairwise differences for (**A**) muscle and (**B**) liver adverse drug reactions (ADRs) among seven statins. Nodes represent statins, with size proportional to the ADR incidence rate (%). Edges represent significant pairwise differences (Tukey-adjusted *p* < 0.05), with thickness proportional to the magnitude of the estimated difference. Arrows indicate the direction of risk, pointing from the statin with significantly higher ADR incidence toward the statin with lower incidence. Edge colors distinguish ADR type: red for muscle ADRs (**A**) and blue for liver ADRs (**B**). Based on arrow directionality, high-risk statins (atorvastatin and pitavastatin) are characterized by predominantly outgoing arrows, while low-risk statins (rosuvastatin and simvastatin) show predominantly incoming arrows.

Network graphs were then constructed to visualize the patterns of significant pairwise differences in ADR incidence as shown in Fig. 3A, B, providing an intuitive summary of the data in Tables 2 and 3. The muscle-ADR network visually reinforced the central role of atorvastatin and pitavastatin (Fig. 3A). Atorvastatin and pitavastatin both show predominantly outgoing arrows, identifying them as high-risk nodes, while rosuvastatin and simvastatin show predominantly incoming arrows, placing them in the low-risk group. In the liver network, pitavastatin emerges as the dominant high-risk node with multiple outgoing arrows, followed by atorvastatin, while rosuvastatin and simvastatin again form the low-risk group (Fig. 3B). The remaining statins (fluvastatin, lovastatin, pravastatin) exhibit mixed or fewer connections in both networks, representing an intermediate-risk category. These network visualizations effectively synthesize the pairwise comparison results, offering a clear, at-a-glance confirmation of the distinct risk hierarchies identified through statistical testing.

## Discussion

This post-hoc HRAF analysis of large-scale Chinese clinical trials delineates distinct and statistically significant safety profiles for muscle and liver ADRs across seven statins. The layered design, moving from global detection to individual identification and finally to pairwise confirmation, enabled us to capture the full complexity of these drug-specific risks. Global tests first confirmed that ADR risks are not a class-wide effect but profoundly drug-specific (both *p* < 0.001). Individual profiling with Bonferroni correction then pinpointed atorvastatin and pitavastatin as the primary drivers of elevated myopathy and hepatotoxicity risks, respectively. Finally, pairwise comparisons with Tukey adjustment not only validated these findings but also quantified the precise risk gradients between all statins, for instance, establishing that pitavastatin’s hepatotoxicity risk was significantly higher than every other statin (all *p* < 0.0001), while atorvastatin’s myopathy risk exceeded that of five comparators. The convergence of evidence across all three tiers, further substantiated by large positive residuals and central positions in network graphs, provides robust reassurance that these risk hierarchies are not artifacts of multiple testing or chance findings. Notably, pitavastatin’s hepatotoxicity profile was particularly striking, demonstrating both statistical significance and clinically substantial magnitude (5.36%; Bonferroni *p* < 0.001). This hierarchical approach addresses key statistical challenges in comparative safety research, controlling for multiplicity while preserving power, and providing progressive validation from global signals to specific pairwise contrasts, ensuring that the resulting risk stratification is both statistically rigorous and clinically actionable.

This risk variability underscores the critical influence of pharmacogenetic and metabolic profiles prevalent in the Chinese population. Consequently, our results advocate for a decisive shift away from a one-size-fits-all approach toward personalized statin selection. By aligning statin choice with individual susceptibility to specific ADRs, such as avoiding high-myopathy-risk atorvastatin in susceptible patients or exercising heightened caution with pitavastatin regarding liver function, clinicians can significantly mitigate treatment-related complications, improve adherence, and optimize long-term cardiovascular outcomes.

This drug-specific risk hierarchy, however, is not reflected in current clinical guidelines, which limited specific recommendations for statin selection based on ADR profiles^6,11,12^. Although statins differ in properties such as potency, solubility, and metabolic pathways^13^, clinical practice often relies on a trial-and-error approach, switching statins only after ADRs occur, rather than employing an initial selection strategy for high-risk patients. This problem is compounded by a scarcity of direct head-to-head comparative studies. Analyses based on spontaneous reporting databases, which yield metrics like the reporting odds ratio (ROR), are inherently limited by a lack of key data, such as reporting rates and population denominators, and are susceptible to geographical reporting biases. To address these critical gaps, our study conducted a meticulous head-to-head analysis of over three decades of clinical trials specifically in the Chinese population. By applying a sample size threshold to mitigate publication bias from small-scale studies, we enhance the robustness of the comparative ADR evidence for this demographic.

Evidence from diverse populations underscores the clinical significance of statin-associated myopathy. A U.S. study reported that statin discontinuation resolved muscle pain in 45 patients, with 13% requiring hospitalization for rhabdomyolysis; however, the specific statin involved was not identified^14^. More specifically, a mechanistic study linked high-dose atorvastatin to impaired mitochondrial function in skeletal muscle, providing a pathophysiological basis for its myopathic potential^15^. However, there is a dearth of direct comparative data on ADRs among statin drugs in the Chinese population^16,17^. Our findings directly build upon this evidence, quantifying the risk in the Chinese population. We not only confirmed cases of rhabdomyolysis (188 among 163,810 patients)^7^ but, through direct comparison, identified atorvastatin as a statin with a significantly higher muscular ADR risk relative to its counterparts in Chinese patients.

The hepatotoxicity risk of atorvastatin identified in our study is consistent with a substantial body of evidence. International pharmacovigilance data consistently identify atorvastatin as the statin most associated with a cholestatic pattern of liver injury^18^, with its top-reported adverse events including specific hepatic manifestations^19^. This concern is echoed within the Chinese population, evidenced by case reports of fatal hepatic failure^20^ and observations that switching from atorvastatin to pravastatin resolved enzyme elevations^21^. The latter study, which noted symptom resolution upon switching to pravastatin, not only aligns with our comparative safety profile but also points to a viable clinical alternative. The convergence of international and local evidence underscores the necessity of incorporating these risk profiles into statin selection for Chinese patients.

Our finding of a significant hepatotoxicity signal for pitavastatin contrasts with a South Korean post-marketing study that reported very low ADR rates^22^. This discrepancy is best explained by fundamental methodological differences. Our analysis, based on clinical trials with active, protocol-defined monitoring, is designed to detect ADRs. In contrast, passive post-marketing surveillance often captures only the most severe events, leading to substantial under-reporting of non-severe but clinically relevant ADRs. The fact that less than 1% rate of significant liver enzyme elevations in that passive surveillance^22^ is itself notable and aligns with the elevated risk we observed. Moreover, a positive signal for pitavastatin-related liver disorders (ROR = 3.34) in the US FDA database^19^ corroborates the existence of a genuine safety concern. Thus, the collective evidence suggests that the risk profile of pitavastatin requires careful attention in Chinese patients, and our findings from rigorously monitored trials offer a critical correction to the potential underestimation in passive surveillance systems.

This study has several limitations, including its basis in clinical trial populations but not real-world data, which may limit generalizability; the inability to adjust for all potential confounders like comorbidities, dosages and concomitant medications; and its focus solely on muscular and hepatic ADRs. Nonetheless, our findings offer crucial insights for personalizing statin therapy in Chinese patients. The identified risk hierarchy, positioning atorvastatin and pitavastatin as higher-risk agents for myopathy and hepatotoxicity, respectively, supports a stratified prescribing strategy. This strategy would involve selecting lower-risk statins (e.g., rosuvastatin or simvastatin) for patients with pre-existing organ-specific vulnerabilities. Consequently, this work underscores the importance of moving beyond a one-size-fits-all model and leveraging head-to-head safety comparisons to optimize patient outcomes.

## Conclusion

This HRAF-based head-to-head comparison provides robust, quantitative safety data for statins in Chinese patients, supporting a shift toward risk-stratified prescribing. The framework identified pitavastatin as warranting heightened caution regarding liver function and atorvastatin as requiring judicious use in myopathy-susceptible patients, while rosuvastatin and simvastatin emerged as lower-risk alternatives for both outcomes. The consistency of findings across global tests, individual profiling, and pairwise confirmation, further reinforced by network visualization, underscores the reliability of this risk hierarchy. Future studies applying this HRAF to real-world data will be valuable for validating its broader utility in evidence-based, personalized pharmacotherapy, providing a critical foundation for investigating the pharmacogenetic mechanisms and potential phenoconversion underlying these differential risks.

## Supplementary Information

Below is the link to the electronic supplementary material.

Supplementary Material 1

## Data Availability

The data supporting the findings of this study are aggregated summary statistics (e.g., event counts and sample sizes) extracted and re-analyzed from our previously published systematic review as described in the Methods. The complete dataset curated for the specific analyses in this manuscript is available from the corresponding author upon reasonable request.

## Abbreviations

ADR: Adverse drug reaction
ALT: Alanine aminotransferase
ANOVA: Analysis of variance
AST: Aspartate aminotransferase
CNKI: China National Knowledge Infrastructure
CVD: Cardiovascular disease
GLM: Generalized linear model
HRAF: Hierarchical Risk Assessment Framework
HSD: Honest significant difference
ROR: Reporting odds ratio

## Declarations

### Patient and public involvement statement

Not applicable. As this study is centered on the analysis of previously published clinical trial data, rather than involving direct interaction with patients or the public in the research design, data collection, or interpretation processes.
