# Supplementary material for: A hierarchical risk assessment framework for head-to-head comparison of statin safety profiles in Chinese patients
Source: Sci Rep. 2026 Apr 9;16:16783. doi: 10.1038/s41598-026-47279-y (PMC13223228; doi:10.1038/s41598-026-47279-y)
Supplement: Supplementary file 1 — Supplementary Material 1 [file 41598_2026_47279_MOESM1_ESM.pdf]

Supplemental Table 1 Standardized residuals for the association between statin treatment and muscle adverse drug reactions (ADRs). Standardized residuals quantify the difference between observed and expected frequencies in each cell of the contingency table. Residuals with absolute values > 2 are considered statistically significant ($p \approx 0.05$), indicating a meaningful deviation from the expected distribution.

Statins	Muscle ADRs	Standardized Residual
Atorvastatin	Positive	9.050111
Atorvastatin	Negative	-9.050111
Rosuvastatin	Positive	-3.884324
Rosuvastatin	Negative	3.884324
Simvastatin	Positive	-5.562738
Simvastatin	Negative	5.562738
Pravastatin	Positive	-2.351468
Pravastatin	Negative	2.351468
Pitavastatin	Positive	3.190883
Pitavastatin	Negative	-3.190883

Supplemental Table 2 Standardized residuals for the association between statin treatment and liver adverse drug reactions (ADRs). Standardized residuals quantify the difference between observed and expected frequencies in each cell of the contingency table. Residuals with absolute values > 2 are considered statistically significant ($p \approx 0.05$), indicating a meaningful deviation from the expected distribution.

**Statins**	**Liver ADRs**	**Standardized Residual**
Atorvastatin	Positive	7.450276
Atorvastatin	Negative	-7.450276
Rosuvastatin	Positive	-6.310541
Rosuvastatin	Negative	6.310541
Simvastatin	Positive	-5.070608
Simvastatin	Negative	5.070608
Pitavastatin	Positive	9.731440
Pitavastatin	Negative	-9.731440
